# Conditioning Conformity: A Neuroscientific Funding and Publishing Paradox

**DOI:** 10.1523/ENEURO.0193-24.2024

**Published:** 2024-05-16

**Authors:** Christophe Bernard

**Affiliations:** Editor-in-Chief, *eNeuro*

Why does groundbreaking progress in our understanding of the brain seem scarce (at least to me)? The prevailing system of research funding and publication, deeply rooted in peer review and contingent upon preliminary data, tends to favor safe, conventional ideas, thereby impeding major breakthroughs. The prerequisite of preliminary data for hypothesis validation transforms grant-seeking into a cycle that prioritizes data accumulation over pioneering ideas, fostering publications that align with pre-established notions endorsed by reviewers. Are we, the scientists, becoming akin to the subjects of our experiments? Consider this: in behavioral studies, rodents are often deprived of food and water to motivate their learning of a task. Since a successful task is rewarded, animals quickly learn the task and become very efficient at it after some time. Now, envision neuroscientists in a similar plight, navigating the pursuit of funding and recognition in high-profile journals. This predicament is faced by many (from undergraduate students to established principal investigators) in our field: a constant scramble for financial support and influential publications to advance our research and careers. But who holds the power to reward our efforts? In the case of rodents, scientists dictate the terms; they design the experiments and determine the rewards. Unlike Douglas Adams’ claim in *The Hitchhiker's Guide to the Galaxy* that humans are monitored by mice (but are we?), it is our human peers who judge the worthiness of our work for funding and publication.

Does this relentless cycle of deprivation and reward truly nurture innovation, or are we conditioning our brightest minds to conform to a well-trodden path? To illustrate my point, consider an analogy to Escher's famous staircase, which we ascend in the hopes of attaining a deeper understanding of the brain (Fig. 1). However, this pursuit is an illusion akin to Escher's endless staircase, where progress seems perpetual but ultimately leads us nowhere close to a real understanding.

**Figure 1.**
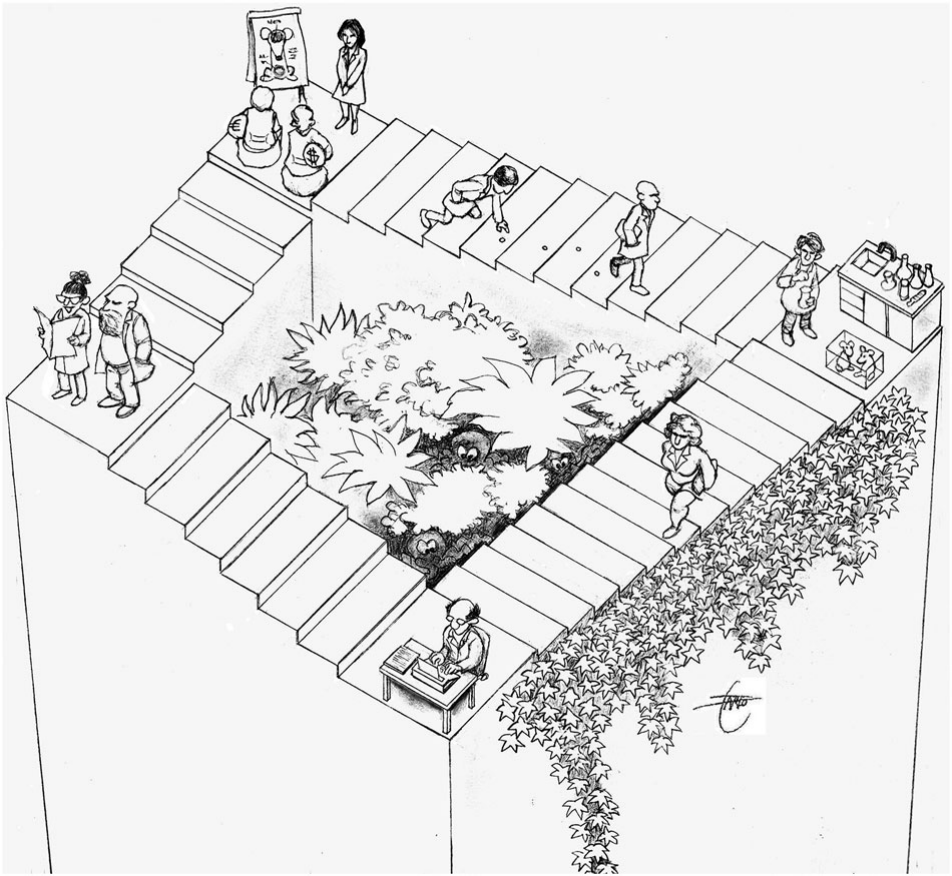
The scientist endless staircase. Courtesy of Laurent Farlotti.

## The Funding and Publication Maze

The current funding model is a maze of its own. If I want my reward, I must have a hypothesis and provide preliminary data, a requirement that ostensibly ensures the feasibility of research and supports the hypothesis. However, the adjudicators of this labyrinth are not distant figures but rather peers within the scientific community, including ourselves. As a result, we learn to conform to the expectations of the community, perpetuating a self-imposed code for success.

Similarly, the publication process mirrors this maze. Here, the elusive reward is not funding but the acceptance of our work in prestigious journals. In this arena too, we embody dual roles—as researchers striving for publication and as peer reviewers bestowing approval. This creates a reciprocal system where the criteria for success are crafted and perpetuated by the community itself.

In our community, all researchers are in constant interaction, in particular the author–reviewer component. The behavior of the community cannot be understood by knowing its individual parts, which aligns to the definition of a complex system. I contend that the present state of the funding and publication maze is an emergent property. We cannot blame a particular component, but that should not prevent us from acting on it.

## The Pavlov–Stakhanov Condition of Researchers

The necessity for preliminary data in grant applications is, in essence, a pavlovian bell, conditioning researchers to pursue a specific hypothesis, leaving little room for the serendipitous discoveries that have historically revolutionized science. This conformity is the antithesis of innovation, a supposed safeguard that instead may be the blind spot in our vision for progress.

Similarly, in behavioral studies, the phenomenon of overtraining animals for efficiency in tasks—intended to optimize data collection—mirrors this conformity. This methodological choice prioritizes animals that excel in specific tasks, akin to selecting grant proposals and research papers based on their alignment with established expectations. This process echoes the basis of the Stakhanovite movement (*стаха́новское движе́ние*), promoting productivity and dedication. However, unlike the Stakhanovite movement, which was directed by the communist party in the Union of Soviet Socialists Republic, this trend in neuroscience research has evolved organically within the community (an emergent property), as a response to the competitive landscape for funding and publication in prestigious journals.

## The Conformity Conundrum

The current funding system exhibits a marked preference for conventional research, often at the expense of ideas that could lead to paradigm shifts. All grants rightfully claim novelty, the raison d’être of research. But this novelty must not be too bold. As a grant writer, I sometimes find myself in an uncomfortable situation, compelled to shape the grant to align with reviewers’ expectations. This necessitates a degree of self-censorship, as I omit mentioning the experiments I think are most exciting but anticipate being labeled as too speculative or lacking preliminary data by the reviewers. Nevertheless, should the grant be awarded, these are precisely the experiments we intend to pursue. This paradox transforms the grant-writing process into a challenging exercise, compounded by the need for self-censorship.

This phenomenon is not confined to grant submissions alone. In my dual roles as an author and reviewer, I have observed a similar bias in publication practices. Novel ideas frequently face rejection from prestigious journals for deviating from mainstream thought, evaluated too avant-garde or unsupported by existing literature. This results in conformity, where unwritten and often subconscious standards dictate what constitutes top-tier research. In addition, the set of authors publishing in high-profile journals are often enlisted as reviewers in the same (and other) journals, further cementing their perspectives as the benchmark for quality and frontier research. As mentioned above, this is not due to a specific will to construct such a system; it is an emergent property.

As a result, the academic community, albeit unconsciously, ends up within a self-imposed echo chamber of ideas, seldom challenging the established schools of thought. Even if you end up in the central courtyard garden where the deep truths are hiding (Fig. 1), presenting divergent viewpoints or challenging the status quo can significantly hinder the publication of one's research.

## The Illusion of Progress

Here I want to challenge the notion that accumulating complex data, particularly via the development of more sophisticated tools, equates to scientific advancement. I remember the time when the gene knock-out technology was introduced. All of a sudden, we were promised a solution to genetic diseases and the unravelling of the mysteries of the brain. At that time, generating a knock-out mouse was a near guarantee to be published in high-impact journals and, by extension, promising academic careers, which strongly biased research axes of the new generations. One can replace knock-out with optogenetics (and many other technological advances) and apply the same reasoning. Have these technological advances translated into a profound understanding of brain function or merely given us the illusion of progress? After all, even when using the most advanced techniques, we still do not understand how a single simple prokaryote cell functions.

The (unspoken) belief that the complexity of the brain eludes us due to inadequate measurement tools might be a misconceived notion. While technologies like all-optical probing and high-field MRI (among many others) contribute vast amounts of data, they have not necessarily culminated in a deeper understanding. The essence of scientific inquiry is not just to accumulate novel data but to produce understanding. Without a theoretical framework akin to Einstein's relativity to guide our exploration of the brain, we find ourselves ascending an endless staircase, rich in novelty but poor in understanding. However, we can now collect vast amounts of data, in particular “brain” maps (e.g., the Allen Institute), that can be used for exploratory research. Such datasets are for the most part hypothesis-free and available to all.

## Breaking the Loop

The French research ecosystem was not relying on competitive grants in the past. French public laboratories would receive a predetermined amount of money every year to do the research they wanted to do, thus creating an environment where taking risks and running hypothesis-free projects was possible. The transition to a centralized grant agency intended to allocate resources to “exciting projects” and stimulate competition has de facto enforced the Pavlov–Stakhanov conditioning among French scientists, valuing conformity over innovation.

I do not know whether the previous system is superior to the current one. As for the review system, if there were an ideal system, it would have been universally adopted by now.

Specialized funding havens like Janelia Research Campus offer financial freedom to a select few. Some funders propose exploratory grants. They should be generalized. However, since we will always (perhaps unconsciously) favor projects that contain strong preliminary data, we need to retrain ourselves to evaluate grants with a different state of mind. As a kid, I was fascinated by explorers, because they went to places where nobody had gone before. I had placed Aldrin, Armstrong, and Collins at the top of my list. My prime motivation for doing a PhD in neuroscience was to imitate them: going into the unknown, the brain, the last frontier of humankind. I wanted to be an explorer. Since when did “exploratory” acquire a negative connotation?

There is a way to break the loop, but our community does not like the idea. We could have a goal-oriented system, in which priorities are decided by external entities, such as finding a cure to migraines. Such a project does not have to be hypothesis-driven but goal-driven, that is, “We do not care about the mechanisms as long as you find something that works.” The mechanisms underlying most neurological disorders remain to be uncovered, yet we have many drugs that can help patients. Most of the time, we do not really know what these drugs do to the brain (and the body). Yet, the fact that they work is all that matters to patients. Whether goal-oriented projects would work is a matter of debate.

In the meantime, for the majority, conducting original research outside grants most often requires obtaining grants on already half-finished projects (the preliminary data that support the hypothesis and feasibility) and using this money to do something that drives us to the core. I confess that my best serendipitous findings were obtained that way. But we don't want funders to know that, do we? This shows how twisted the system can be. We are all aware of it, but if we want to survive, we need to play by the rules. Just like for the publication of our results, we all know that the impact factor is not a good metric for research quality/impact, but we still play the game, because if we do not play it, we will not survive.

## Conclusion: A Call for Reflection and Change

Many voices have highlighted the limitations of the models used to produce knowledge, finance research, and publish it over many decades. I am just repeating what has already been told before. You may notice that these essays always conclude that the neuroscience community stands at a crossroads, equipped with the tools of innovation yet constrained by the very structures that govern our research. Why have we been staying at crossroads for decades, staying on the same endless staircase? Perhaps, because the latter gives us the comforting illusion of climbing the stairs of understanding.

It is easy to say: embrace the uncertainty of exploration, champion theoretical development, and cultivate a funding and publication ecosystem that rewards not just the quantity of data, but the quality of insights. They remain empty words, if no concrete action is taken. Let us remember that the rules of engagement were not imposed from without but emerged from within our community. This editorial will not change the status quo but may hopefully plant some seeds. The first step is to be aware of the problem, before rethinking these self-imposed constraints. Being overly optimistic, I still believe that I will still be alive when a major paradigm shift regarding brain function is made.

